# Non-literacy biased, culturally fair cognitive detection tool in primary care patients with cognitive concerns: a randomized controlled trial

**DOI:** 10.1038/s41591-024-03012-8

**Published:** 2024-06-04

**Authors:** Joe Verghese, Rachel Chalmer, Marnina Stimmel, Erica Weiss, Jessica Zwerling, Rubina Malik, David Rasekh, Asif Ansari, Roderick A. Corriveau, Amy R. Ehrlich, Cuiling Wang, Emmeline Ayers

**Affiliations:** 1 Departments of Neurology, Albert Einstein College of Medicine, Bronx, NY USA; 2 Department of Epidemiology and Population Health, Albert Einstein College of Medicine, Bronx, NY USA; 3 Department of Medicine, Montefiore Medical Center, Bronx, NY USA; 4 Department of Neuroscience, National Institute of Neurological Disorders and Stroke, Bethesda, MA USA

**Keywords:** Randomized controlled trials, Physical examination, Alzheimer's disease

## Abstract

Dementia is often undiagnosed in primary care, and even when diagnosed, untreated. The 5-Cog paradigm, a brief, culturally adept, cognitive detection tool paired with a clinical decision support may reduce barriers to improving dementia diagnosis and care. We performed a randomized controlled trial in primary care patients experiencing health disparities (racial/ethnic minorities and socioeconomically disadvantaged). Older adults with cognitive concerns were assigned in a 1:1 ratio to the 5-Cog paradigm or control. Primary outcome was improved dementia care actions defined as any of the following endpoints within 90 days: new mild cognitive impairment syndrome or dementia diagnoses as well as investigations, medications or specialist referrals ordered for cognitive indications. Groups were compared using intention-to-treat principles with multivariable logistic regression. Overall, 1,201 patients (mean age 72.8 years, 72% women and 94% Black, Hispanic or Latino) were enrolled and 599 were assigned to 5-Cog and 602 to the control. The 5-Cog paradigm demonstrated threefold odds of improvement in dementia care actions over control (odds ratio 3.43, 95% confidence interval 2.32–5.07). No serious intervention-related adverse events were reported. The 5-Cog paradigm improved diagnosis and management in patients with cognitive concerns and provides evidence to promote practice change to improve dementia care actions in primary care.

ClinicalTrials.gov: NCT03816644.

## Abstract (web summary)

The 5-Cog paradigm, a culturally fair and non-literacy biased cognitive detection tool paired with a clinical decision support improved cognitive impairment diagnosis and management threefold in primary care patients with cognitive concerns.

## Main

Dementia is common among older adults, with one in nine older Americans affected, and rates are increasing worldwide^1^. Despite the availability of many cognitive assessment tools, dementia is often undiagnosed; over half of dementia cases are missed in primary care^2–4^. This problem is more prevalent among older Black and Hispanic people than older white people in the United States^4–6^. Barriers to implementing routine cognitive detection and related care actions in primary care are at the level of the instrument, patient, clinician and healthcare system^2,3,5,7–11^. Many cognitive detection or diagnosis approaches are long, expensive, require clinicians to administer, need specialized equipment or do not provide guidance on the next steps following normal or abnormal results^5,9,12–14^. Over a quarter of US residents belong to an ethnic minority such as Black, Hispanic or Latino (https://www.census.gov/quickfacts/fact/table/US/PST045223), but many cognitive tests were developed in white populations^6,15^. These tests, therefore, do not adequately account for cultural differences or health inequity^5,6,15^. For example, we reported that the Montreal Cognitive Assessment (MoCA) test cutoff points for detecting dementia that were established in mostly white populations were too high in our US-based study sample that consisted of mostly Black or Hispanic people^16^. Black and Hispanic participants in the US-based Health and Retirement study were reported to have missed or delayed diagnosis of dementia more often compared to white people^17^.

In the US, primary care providers (PCPs) are clinicians (mostly physicians and sometimes nurse practitioners) who practice general medicine and have the key responsibility for dementia diagnosis and care^2,18,19^. PCPs are positioned to make a timely diagnosis of dementia as they have more frequent contacts as well as long-term relationships with patients that engender trust^20^. Cognitive assessment is well accepted by patients when endorsed by their PCPs^3,8,13,19,21^. Hence, cognitive detection with recommended follow-up actions based on test outcomes, and referral if necessary to specialists, may reduce barriers to dementia diagnosis and care in primary care^3,21^. There have been many primary care-based studies that have examined the validity of brief cognitive assessments^3,5,9,13,19^, physician education^22^ or dementia care management^10,23^ to improve care of cognitively impaired patients. But these dementia care elements have been mostly studied in isolation and there is insufficient evidence to conclude which approach may improve dementia care in primary care settings^10^, especially in populations experiencing health disparities. To overcome current limitations, we developed the 5-Cog paradigm, a non-literacy biased, culturally fair and easy-to-use 5-min cognitive assessment paired with an electronic medical record (EMR)-embedded clinical decision tree to assist PCPs to make dementia care choices. We performed a single-blind randomized controlled trial (RCT) to test the efficacy of the 5-Cog paradigm to improve dementia care actions related to diagnosis, investigation and management of older primary care patients with cognitive concerns.

## Results

### Patient disposition

**Fig. 1:**
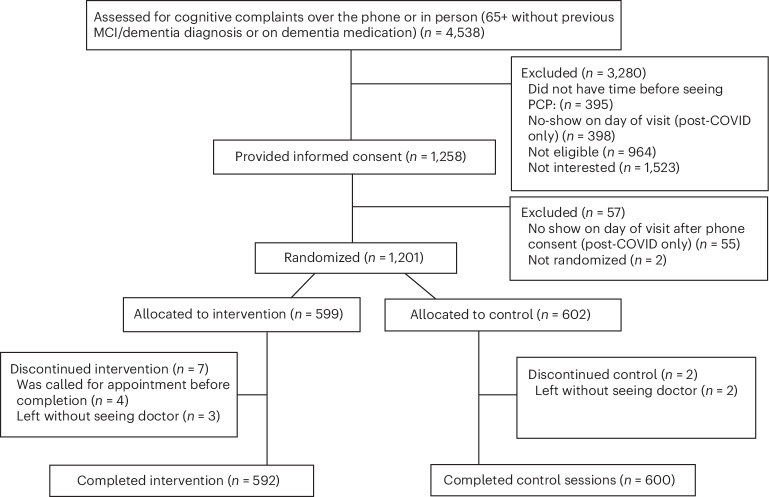
Study flow. Flow diagram for study participants.

Of 4,538 patients screened for eligibility, 1,258 with cognitive concerns were deemed eligible and provided consent, and 1,201 participants were enrolled from 29 May 2019 to 15 September 2022 (Fig. 1), 599 assigned to 5-Cog and 602 to control. In all, nine participants withdrew from the study (5-Cog, *n* = 7; control, *n* = 2). Follow-up for the primary outcome ended on 15 December 2022, and on 15 May 2023 for the secondary outcome. The outcome ascertainment rate was 100% in both arms.

Of the 1,201 participants with cognitive concerns, the median age was 72.8 years (range 65–98), 865 (72.0%) were women and 485 (40.4%) did not graduate from high school. All participants (100%) resided in zip codes designated as socioeconomically disadvantaged neighborhoods (high Area Deprivation Index^24^). Overall, 585 (48.7%) participants self-reported their race as Black and 667 (55.5%) reported ethnicity as Hispanic or Latino. A total of 681 (56.7%) participants were assessed in English and 520 (43.3%) in Spanish. Baseline characteristics were well balanced across arms (Table 1).

**Table 1:** Baseline characteristics of intervention and active control arms

Variables	Intervention (*N* = 599)	Active control (*N* = 602)
Age, years (mean ± s.d.)	72.88 ± 6.38	72.64 ± 6.67
Sex		
Female, *n* (%)	431 (72.0)	434 (72.1)
Male, *n* (%)	168 (28.0)	168 (27.9)
Race or ethnic group, *n* (%)		
Black	305 (50.9)	280 (46.5)
White	59 (9.8)	59 (9.8)
Asian	13 (2.2)	10 (1.7)
American Indian/Alaskan native	4 (0.7)	4 (0.7)
Hawaiian	2 (0.3)	1 (0.2)
More than one race	33 (5.5)	28 (4.7)
Other	29 (4.8)	38 (6.3)
Not reported/refused	154 (25.7)	182 (30.2)
Hispanic or Latino ethnic group, *n* (%)	323 (53.9)	344 (57.1)
Education		
Years of education, mean ± s.d.	11.24 ± 4.10	10.95 ± 4.26
Some high school or less, *n* (%)	232 (38.7)	253 (42.0)
High school graduate or higher, *n* (%)	367 (61.3)	349 (58.0)
Socioeconomic disadvantaged neighborhood residency, *n* (%)	100	100
Language of test administration, *n* (%)		
English	339 (56.6)	342 (56.8)
Spanish	260 (43.4)	260 (43.2)

Of the 63 patients diagnosed with mild cognitive impairment (MCI) or dementia in the 5-Cog arm, 56 (89%) received the diagnosis on the day of the visit, 6 by day 30 and 1 by day 60. Of the 12 control patients diagnosed with MCI or dementia, 11 (92%) received the diagnosis on the day of the visit and 1 by day 30. In the intervention arm, the 5-Cog battery had a sensitivity of 96% and specificity of 71% for PCP diagnoses of MCI or dementia, suggesting a low false-negative rate. In 295 participants in the 5-Cog arm who agreed to additional cognitive testing after seeing their PCP, the 5-Cog battery had a sensitivity of 70% and specificity of 71% for detecting MCI or dementia using a cutoff score of <17 on the MoCA test^2,16^.

### Primary outcome

**Fig. 2:**
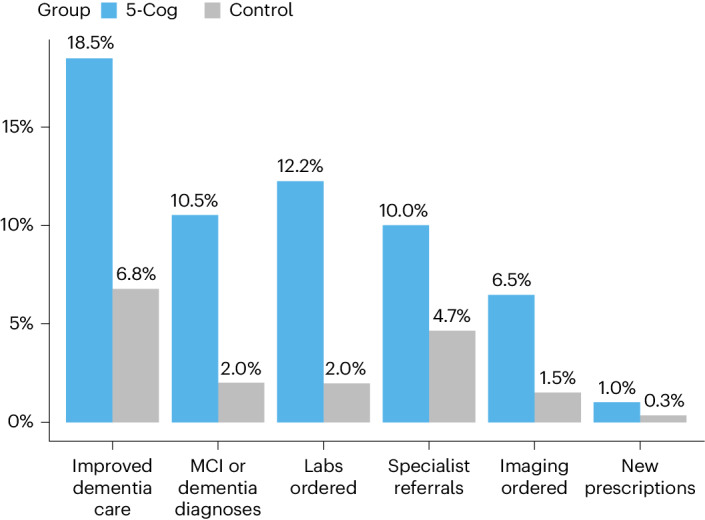
Outcome rates by study arm. Proportion of participants in the 5-Cog (blue) and control (grey) study arms who met improved dementia care outcome as well as individual criterion by 90 days.

Dementia care actions were higher in the 5-Cog arm (18.5% versus 6.8%, *P* < 0.001). The adjusted odds ratios (ORs) for the primary outcome (OR 3.43) and individual components (OR 2.38–7.64) revealed higher rates in the 5-Cog arm than the control (Table 2). Dementia care actions were seen in 106 (43.8%) out of 242 patients with positive 5-Cog results and only in 5 (1.4%) with negative 5-Cog. New PCP diagnoses of dementia (3.5% versus 1.5%) and MCI (7.3% versus 0.8%) were higher in the 5-Cog arm. New prescriptions were rare in both arms (1.0% versus 0.3%, *P* = 0.15). Figure 2 compares outcomes by arm.

**Table 2:** Improved dementia care actions related to diagnosis, investigation and treatment

Outcome	5-Cog *n* (%) (*N* = 599)	Active control *n* (%) (*N* = 602)	OR (95% CI)^a^	*P* value
Improved dementia care	111 (18.5)	41 (6.8)	3.43 (2.32–5.07)	<0.001
New MCI or dementia diagnoses	63 (10.5)	12 (2.0)	6.48 (3.41–12.31)	<0.001
Imaging ordered	39 (6.5)	9 (1.5)	4.80 (2.29–10.06)	<0.001
Tests ordered	73 (12.2)	12 (2.0)	7.64 (4.05–14.39)	<0.001
New prescriptions	6 (1.0)	2 (0.3)	3.24 (0.63–16.65)	0.15
Specialist referral	60 (10.0)	28 (4.7)	2.38 (1.49–3.80)	<0.001

^a^Adjusted for age, sex and education. CI, confidence interval.

### Secondary outcome

A total of 1,042 participants (86.8%) completed 12 months of follow-up. There were no statistically significant group differences in hospitalizations (5-Cog 15.2% versus control 16.7%, *P* = 0.49) or emergency department visits (16.3% versus 15.2%, *P* = 0.61) over 12 months.

### Safety

No study-related adverse events (such as medical symptoms or signs, anxiety or depressive symptoms) were observed by research or clinic staff. No notes indicating possible harm were recorded by clinicians in the EMR during the 90-day observation period.

### Sensitivity analyses

Primary outcome results were statistically significant in subgroups defined by sex, ethnicity, education and language (Table 3). The 5-Cog arm had more improved dementia care actions than the control in all comparisons.

**Table 3:** Sensitivity analyses of primary outcome (improved dementia care actions) in subgroups

Groups	Subgroups	*N*	OR (95% CI)^a^
Sex	Male	336	2.21 (1.13–4.35)
	Female	865	4.23 (2.60–6.86)
Ethnicity	Black	585	3.57 (1.90–6.70)
	Hispanic	667	2.94 (1.83–4.73)
Language tested	English	681	4.26 (2.29–7.93)
	Spanish	520	2.94 (1.76–4.91)
Education	less than high school	485	3.56 (2.16–5.88)
	high school graduate and higher	716	3.20 (1.71–5.97)
COVID-19	Pre-suspension	457	4.02 (2.20–7.36)
	Post-suspension	744	3.11 (1.86–5.22)

^a^Adjusted for age, sex and education years.

### Post-hoc analysis

Participants who enrolled before and after COVID-19 suspension were similar in baseline characteristics (Supplementary Table 1) and primary outcome (Table 3).

## Discussion

In this single-blind RCT, the 5-Cog paradigm (brief cognitive assessment paired with an EMR-embedded clinical decision tree) demonstrated threefold odds of improvement in dementia care actions in older primary care patients with cognitive concerns over an active control. The 5-Cog paradigm was brief and well accepted as per patient and staff feedback. Excellent validity of numerous brief cognitive assessments (including PMIS^25,26^) for detecting dementia is reported^3,5,9,13,19^, but a lack of evidence for their effect on patient care outcomes remains^11,27^. In 2020, the US Preventive Task Force (USPTF) concluded that there was insufficient evidence to assess the benefits and harms of systematic cognitive assessment in asymptomatic older adults^11^. Early case detection through assessment of cognitive signs and symptoms (as in this trial^2^) was not considered as screening by the USPTF and was not addressed^11^. Hence, our findings, based on cognitive assessment triggered by cognitive concerns, not only tackle a major gap in the USPTF recommendations^11^ but also address pragmatic considerations of medical care for people experiencing cognitive decline. This large-scale RCT establishes the efficacy of a brief cognitive detection paradigm to improve dementia care actions related to diagnosis, investigation or treatment in a primary care population experiencing health disparities in the United States.

Individual dementia care actions were much higher in the 5-Cog arm except for new prescriptions: 5-Cog participants received more MCI or dementia diagnoses (OR 6.5) as well as laboratory (OR 7.6) or imaging tests (OR 4.8), and specialist referrals (OR 2.4) for cognitive indications than controls. Some reasons for the low prescription rate may include awaiting investigations or specialist evaluations, inexperience in managing dementia or concerns about effectiveness and side effects of medications.

PCPs used the 5-Cog paradigm to aid decision-making but seemed to rely on their own clinical judgment; not initiating actions in most patients with abnormal results. There was a much lower rate of dementia care actions in test-negative 5-Cog and control patients, even though PCPs were aware that enrolled patients had cognitive concerns, received education regarding dementia management and had access to cognitive tests, including the picture-based memory impairment screen (PMIS) in the EMR^2,25^. The low diagnosis and action rates in the control arm are in line with primary care estimates^3,4,21,28^ and suggests that PCPs were not primed by the education provided to make more diagnoses or actions than routine. A previous unblinded RCT of an educational intervention designed to improve knowledge and management of dementia in general practice did not alter management or case identification^22^. These findings support the value of the EMR-embedded decision tree in the 5-Cog process. A non-randomized trial of assessment by medical assistants coupled with physician education reported new actions (dementia diagnoses, prescriptions and specialist referrals) in 17% of older patients with positive outcomes for cognitive testing in two intervention clinics compared to 1% in two control clinics^3,21^.

Historically marginalized populations are disproportionately affected by dementia^29–31^ but are underrepresented in RCTs^32^. In our sample, 94% of people were Black or Hispanic, and all lived in socioeconomically disadvantaged neighborhoods in Bronx County in the United States^29–32^. Disparities in dementia care actions are reported with Hispanic people receiving less care than Black or white patients in the United States^20^. The 5-Cog paradigm helped to lessen these inequities; the 5-Cog intervention significantly predicted the primary outcome in Black and Hispanic patients as well as by sex, education and language. The selection of test items for 5-Cog that account for cultural differences and education as well as ease of administration may help mitigate barriers.

PCPs at our busy clinic preferred to receive results and recommendations, rather than administer tests themselves^33,34^. PCPs spend 15–20 min with patients addressing a median of six medical issues^34^, with increased documentation requirements and stress since the introduction of EMR^33,35^; however, the 5-Cog paradigm requires few resources and can be administered by non-physician personnel with minimal training^25,26^. Given that we enrolled only adults with cognitive concerns, the 5-Cog sensitivity and specificity is reasonable. In comparison, in the 5-Cog subsample that agreed to additional testing, we reported that the MoCA test (<17) had a sensitivity of 65% for English and 64% for Spanish versions, and specificity of 77% for English and 73% for Spanish versus the neuropsychologist’s independent diagnosis of cognitive impairment^2,16^. Unlike cognitive assessments with higher stated sensitivity and specificity that were validated in samples with cognitively healthy controls^5,9,13^, test-negative cases in the 5-Cog arm were not cognitively normal as they had cognitive concerns^36^. The 5-Cog recommendation in test-negative patients is to repeat assessment annually. Hence, false-negative cases can be detected at follow-up visits. Our goal was to test the efficacy of the overall 5-Cog paradigm and not the individual components. The 5-Cog battery and recommendation components can be modified based on clinical setting and needs as well as when there are advances in knowledge and treatments for dementia. The applicability of the 5-Cog approach to other cognitive tests can also be examined. The Medicare annual wellness visit requires cognitive assessment^37^ and presents an opportunity to implement the 5-Cog paradigm in primary care clinics across the country. The emergence of new Alzheimer’s therapies further underscores the need for the 5-Cog paradigm to triage and manage primary care patients with cognitive concerns.

Only one trial has reported on the utility of routine systematic cognitive screening (there was no requirement for cognitive concern) in older primary care patients without a diagnosis of cognitive impairment. The outcome of that trial showed no differences at 12 months in quality of life, mood and healthcare utilization^38,39^. A low test positivity rate and fewer evaluations in test-positive cases were noted as limitations of this trial^27^. Our RCT, which required there to be a cognitive concern identified to proceed with the 5-Cog evaluation, did not increase hospitalization or emergency department visits. Only two healthcare metrics (hospitalizations and emergency department visits) were examined as the secondary outcome, but we are planning a detailed economic analysis. A minority of patients (10%) in the 5-Cog arm were referred to specialists and did not overwhelm the system^40,41^.

Our trial had certain strengths and many pragmatic elements. First, a preliminary study in our clinic reported a high prevalence of cognitive concerns, up to 40%; highlighting the need for the 5-Cog paradigm^2,42^. Second, racial/ethnic minorities accounted for only one-quarter of participants in a systematic review of 42 US-based dementia clinical trials^29–32^. We directly addressed this health inequity through recruitment of underserved racial/ethnic minorities in the United States^32^. Third, our RCT was reflective of real-world conditions: it was embedded in a primary care clinic and assessments were conducted in English and Spanish without PCPs^2^. Fourth, the actions are relevant to evaluating investigation and management of dementia^3,18,37,43^. Hence, our study addresses the dearth of RCTs that have examined efficacy of cognitive detection on outcomes related to patient care^10,44^. Finally, observed effect sizes in this clinical trial were larger than the original estimates from our power analysis^2^.

Some limitations are noted. The consent procedure (but not the protocol) was changed following COVID-19 suspension, but participants recruited before and after had similar characteristics and results. Given the semi-pragmatic nature of the trial, MCI and dementia classifications were based on PCP diagnoses and not on neuropsychological evaluation, which takes over an hour, and is neither routine nor practical for detecting cognitive impairment in primary care. This approach is reflective of the real world; over 80% of dementia diagnosis among Medicare beneficiaries in the United States were made by non-dementia specialists^20^. We selected dementia care actions from previous studies^3,21,28,43^, but other dementia care quality indicators should also be examined. Many participants did not provide information on race, limiting ethnic subgroup comparisons. Our results do not apply to asymptomatic older patients^11,44^, but the utility of 5-Cog should be determined in this population. A detailed psychological interview was not feasible given the study design but may have revealed stress caused by diagnosis that was not brought to medical attention. We restricted primary outcome ascertainment to 90 days based on our survey. The majority of cognitive diagnoses in both arms occurred on the day of the visit; very few were made after the first month. Cognitive concerns may have multiple etiologies. Hence, the recommendations in both arms suggested evaluation for potentially contributing conditions such as depression, anxiety and medical conditions^2^. Our efficacy trial was based in a single center in the United States and should be examined in other ethnicities and populations worldwide; however, the busy primary care setting is typical of urban clinics in the United States. Furthermore, individual 5-Cog elements have been implemented in many settings in the United States and in resource-poor settings globally^25,26,45^, supporting generalizability. Following up on this clinical efficacy trial, we have begun a pragmatic cluster randomized trial that will examine the clinical effectiveness of the 5-Cog paradigm, including the critical aspect of integration of results with recommendations for follow-up in the EMR, in 22 primary care clinics as well as evaluate implementation issues and economic impact (NCT05515224).

The 5-Cog paradigm helped to improve dementia care actions related to diagnosis, investigations and treatments that account for many implementation barriers and racial/ethnic differences in primary care patients presenting with cognitive concerns. This trial provides the evidence base to develop cognitive detection guidelines to promote practice change in primary care.

## Methods

### Study design

We conducted a single-blind RCT of the 5-Cog paradigm in primary care patients with cognitive concerns (NCT0381664). The study design was guided by pilot studies and feedback from local PCPs^2,42^. The Einstein institutional review board approved the study protocol. Following research suspension during the COVID-19 pandemic (March 2020 to October 2020), the institutional review board amended the informed consent procedure from written to oral (telephone before visit)^2^. No other amendments were made to the protocol^2^.

The main inclusion criteria were age 65 years or more, presence of cognitive concerns, having a clinic appointment and speaking English or Spanish. The main exclusion criteria were previous dementia or MCI diagnoses, being a nursing home resident and inability to see or hear well enough to complete assessments. The full study criteria are in our protocol paper^2^.

### Recruitment

Participants were recruited from one urban primary care clinic in Bronx County, New York, serving adults experiencing health disparities (underserved racial/ethnic minorities and residing in socioeconomically disadvantaged neighborhoods)^2,24^. All 18 PCPs (16 physicians and 2 nurse practitioners) at this site participated in the trial. Research staff reviewed daily clinic schedules to identify potential participants, who were asked whether they or their loved ones were concerned about their memory function^2^. Patients who answered affirmatively to either of the two cognitive concern questions were considered eligible for this trial (98.9%). A minority were recruited via patient self-referrals (0.4%) or staff referrals (0.7%).

### Randomization

The study statistician generated a block randomization sequence stratified by sex and age (< or ≥80 years) using SAS PROC PLAN (SAS Institute Software) to assign participants 1:1 into 5-Cog or control arms. The age cutoff was changed from 75 years to 80 years before the study started to be consistent with widely used definitions of the oldest age group (≥80 years)^46^. Staff were blinded to randomization assignment of the next participant until assigned. Investigators, assessors and statisticians were blinded to individual assignments and did not participate in recruitment or interventions.

### Intervention

Participants randomized to the intervention arm received the 5-Cog battery: PMIS^25,26^, Motoric Cognitive Risk Syndrome (MCR) diagnosis^47,48^ and Symbol-Match^2,49^. While other cognitive tests are available^3,5,9,14^, they were not suitable for our purpose as they did not integrate into the EMR with recommended follow-up actions based on test outcomes. Many tests also do not account for ethnic cultural differences, are lengthy or cannot be administered by non-clinicians^3,5,9,14^. In addition, 5-Cog tests were validated in our local populations^2,25,26,48^. Details of the 5-Cog battery are published^2^. In brief, PMIS is a four-picture free and cued recall memory test that minimizes educational bias. MCR is characterized by cognitive concerns and slow gait^48^. Symbol-Match requires participants to match symbols to corresponding numbers^49^. MCR and Symbol-Match tap into non-memory domains^47–49^.

The tester administered the 5-Cog battery before the patient saw their PCP. If there was not sufficient time to complete the battery before seeing their PCP, the patient was not tested (Fig. 1). After administering 5-Cog, the tester created a note in the EMR including results (‘positive’ or ‘negative’) with a corresponding decision tree containing a set of recommendations based on dementia care guidelines worldwide^50^. The 5-Cog battery was positive if any one test was abnormal. The tester gave the patient a paper token noting study participation, which they presented to their PCP at the same visit. The token reminded the PCP to read the 5-Cog results and recommendations when they opened their patient’s EMR. The EMR note clarified that the 5-Cog results are ‘not diagnostic’ but indicated that the patient is ‘at increased risk’ (positive) or ‘at lower risk’ (negative) of cognitive impairment. Based on PCP feedback^2^, sensitive cutoff scores were selected to minimize false-negative results (fewer cases of disease missed) in this sample of patients with cognitive concerns, a high risk for dementia group^36^.

### Control

The active control matched for time and tester exposure. Elements were chosen to be clinically relevant but not overlap with the 5-Cog battery and included the Short Assessment of Health Literacy in Medicine (SAHL) and grip strength (Jamar dynamometer)^2,51^. SAHL has good reliability and validity in English and Spanish^51^. Grip strength is a validated health and function indicator^2^. Established cutoff scores for low health literacy and weak grip were employed to determine ‘positive’ or ‘negative’ performance^2^. Procedures mirrored those in the 5-Cog arm, including an EMR note with recommendations for ‘positive’ or ‘negative’ results^2^.

PCPs were not required to follow recommendations in both arms and were instructed to use their clinical judgment to decide on management^2^. The full set of recommendations for both arms is provided in our protocol paper^2^. As all patients had cognitive concerns, decision trees in both arms recommended functional assessments and including caregivers in care plans^2^.

### Procedures

Workshops were conducted before the trial to educate PCPs and staff at the clinic about study procedures, MCI/dementia diagnoses and management, frailty and health literacy. Periodic newsletters updated staff about the study.

### Outcomes

The primary outcome was improved dementia care actions related to diagnosis and management of cognitive impairment, a composite outcome met by documentation in EMR of any of the following within 90 days of visit: new PCP diagnoses of MCI or dementia; laboratory or imaging tests; new dementia medication prescriptions or specialist referral for dementia evaluation^2^. These dementia care actions are accepted as important by experts and included in quality of care for dementia studies^3,18,37,43,52^. The 90-day period was chosen as surveys in our site indicated that most dementia care actions were initiated within a week of the visit. The date of the action ordered and not the date completed was taken. Tests, referrals or medications in our EMR can only be ordered by entering a medical indication. Investigations, referrals or treatments were only counted as outcomes if a cognitive diagnosis (for example, MCI) was entered as the indication. Any action ordered for non-cognitive medical indications were not counted toward outcomes. PCPs also made other cognitive diagnoses such as ‘cognitive impairment’, ‘cognitive deficit’ or ‘memory loss,’ in both the 5-Cog and control arms (6.7% versus 3.7%, *P* = 0.02). But these other cognitive diagnoses that do not have standard definitions were not a priori included in the diagnosis criterion for the primary outcome to minimize heterogeneity and ambiguity; however, they were considered as cognitive indications for the other end points (investigations, treatment or referrals) used to define the primary outcome.

Concerns that patients diagnosed with dementia have higher healthcare utilization and costs has been raised^40,41^. Hence, healthcare utilization (emergency room visits and hospitalizations for any reason) up to 12 months after enrollment was examined as a secondary outcome^2^.

Research staff documented adverse events from direct observation as well as feedback from clinic staff during and after the visit. An independent assessor (M.S.), blinded to group assignments, reviewed participants’ EMR for primary (cognitive indications) and secondary outcome end points. The assessor also reviewed EMR notes for documentation of any harm possibly related to the intervention (such as medical symptoms, stress or anxiety). Any ambiguity in clinical documentation was discussed with other experienced clinicians in the team (blinded to group) and resolved by consensus.

### Statistical analysis

The statistical analysis plan was prespecified by the statistician (C.W.) before being unblinded to data. Analyses were performed using SPSS v.29. With 1,200 participants (600 per arm) and assuming a noncompletion rate of EMR entries of 10% and that 25% of controls would experience ‘improved dementia care’, we estimated to detect ORs of up to 1.46 on the effect of the 5-Cog paradigm on improving dementia care with 80% power at an alpha level of 5% (ref. ^2^). According to the intention-to-treat principle, all randomized participants were included in the analysis according to group assigned irrespective of intervention received. Baseline characteristics were summarized with descriptive statistics and compared between groups with a chi-squared test for categorical variables and independent *t*-test for continuous variables. Discriminant validity of the 5-Cog battery at chosen cutoff scores was assessed by computing sensitivity and specificity for detecting PCP diagnosis of dementia or MCI in the 5-Cog arm. Primary and secondary outcomes were compared between arms using chi-squared tests and multivariable logistic regression adjusted for age, sex and education. Chronic illness was originally proposed as a covariate, but this information was not systematically collected and so this variable was not included as a covariate in the analysis. Prespecified sensitivity analyses were conducted to account for heterogeneity of treatment effect due to sex, ethnicity, language of administration and education. We conducted post-hoc comparisons of participants enrolled before and after the COVID-19 suspension using descriptive statistics as described for the primary outcome.

### Reporting summary

Further information on research design is available in the Nature Portfolio Reporting Summary linked to this article.

## Online content

Any methods, additional references, Nature Portfolio reporting summaries, source data, extended data, supplementary information, acknowledgements, peer review information; details of author contributions and competing interests; and statements of data and code availability are available at 10.1038/s41591-024-03012-8.

### Supplementary information

Supplementary Information Supplementary Table 1.

Reporting Summary

## Data Availability

The data that support the findings of this study are not openly available due to reasons of confidentiality. Upon reasonable request, individual deidentified participant data (including data dictionaries) will be made available via a RedCap web-based database, after review and approval of a methodologically sound proposal, with a signed data access agreement, in line with Ethics Committee requirements. Please contact corresponding author, J.V. (joe.verghese@einsteinmed.edu). These files will be available from the date of publication until the date stated in the approved request. The study protocol is available as an open access publication.
