# Supplementary material for: Non-literacy biased, culturally fair cognitive detection tool in primary care patients with cognitive concerns: a randomized controlled trial
Source: Nat Med. 2024 Jun 4;30(8):2356–61. doi: 10.1038/s41591-024-03012-8 (PMC11333278; doi:10.1038/s41591-024-03012-8)
Supplement: Supplementary file 1 — Supplementary Table 1. [file 41591_2024_3012_MOESM1_ESM.pdf]

nature medicine

Article

https://doi.org/10.1038/s41591-024-03012-8

Non-literacy biased, culturally fair cognitive detection tool in primary care patients with cognitive concerns: a randomized controlled trial

In the format provided by the authors and unedited

Supplementary table 1. Baseline characteristics of participants enrolled before and after the COVID-19 research suspension.

Supplementary table 1. Baseline characteristics of participants enrolled before and after the COVID-19 research suspension.

Variables	Pre-Covid (N = 457)	Post-Covid (N = 744)
Age, years	73.30 ± 6.54	72.42 ± 6.50
Sex		
Female, n (%)	331 (72.4)	534 (71.8)
Male, n (%)	126 (27.6)	210 (28.2)
Race or ethnic group, n (%)		
Black	177 (38.7)	408 (54.8)
White	27 (5.9)	91 (12.2)
American India/Alaskan Native	2 (0.4)	6 (0.8)
Hawaiian	1 (0.2)	2 (0.3)
More than one race	25 (5.5)	36 (4.8)
Other	11 (2.4)	56 (7.5)
Not reported	206 (45.1)	130 (17.5)
Hispanic or Latino ethnicity, n (%)	272 (59.5)	395 (53.1)
Years of education, mean SD	10.74 ± 4.16	11.31 ± 4.18
Socioeconomic disadvantaged neighborhood residency, n (%)	100	100
Language of test administration, n (%)		

English	232 (50.8)	449 (60.3)
Spanish	225 (49.2)	295 (39.7)
