# Supplementary material for: Non-literacy biased, culturally fair cognitive detection tool in primary care patients with cognitive concerns: a randomized controlled trial
Source: Nat Med. 2024 Jun 4;30(8):2356–61. doi: 10.1038/s41591-024-03012-8 (PMC11333278; doi:10.1038/s41591-024-03012-8)
Supplement: Supplementary file 2 — Reporting Summary [file 41591_2024_3012_MOESM2_ESM.pdf]

nature portfolio

Corresponding author(s): Joe Verghese

Last updated by author(s): 11/20/2023

# Reporting Summary

Nature Portfolio wishes to improve the reproducibility of the work that we publish. This form provides structure for consistency and transparency in reporting. For further information on Nature Portfolio policies, see our Editorial Policies and the Editorial Policy Checklist.

## Statistics

For all statistical analyses, confirm that the following items are present in the figure legend, table legend, main text, or Methods section.

| n/a | Confirmed | |
|---|---|---|
| ☐ | ☒ | The exact sample size (*n*) for each experimental group/condition, given as a discrete number and unit of measurement |
| ☒ | ☐ | A statement on whether measurements were taken from distinct samples or whether the same sample was measured repeatedly |
| ☐ | ☒ | The statistical test(s) used AND whether they are one- or two-sided<br>*Only common tests should be described solely by name; describe more complex techniques in the Methods section.* |
| ☐ | ☒ | A description of all covariates tested |
| ☐ | ☒ | A description of any assumptions or corrections, such as tests of normality and adjustment for multiple comparisons |
| ☐ | ☒ | A full description of the statistical parameters including central tendency (e.g. means) or other basic estimates (e.g. regression coefficient) AND variation (e.g. standard deviation) or associated estimates of uncertainty (e.g. confidence intervals) |
| ☐ | ☒ | For null hypothesis testing, the test statistic (e.g. *F*, *t*, *r*) with confidence intervals, effect sizes, degrees of freedom and *P* value noted<br>*Give P values as exact values whenever suitable.* |
| ☒ | ☐ | For Bayesian analysis, information on the choice of priors and Markov chain Monte Carlo settings |
| ☒ | ☐ | For hierarchical and complex designs, identification of the appropriate level for tests and full reporting of outcomes |
| ☒ | ☐ | Estimates of effect sizes (e.g. Cohen's *d*, Pearson's *r*), indicating how they were calculated |

*Our web collection on statistics for biologists contains articles on many of the points above.*

## Software and code

Policy information about availability of computer code

| | |
|---|---|
| Data collection | Redcap |
| Data analysis | SPSS v. 29; SAS PROC PLAN |

For manuscripts utilizing custom algorithms or software that are central to the research but not yet described in published literature, software must be made available to editors and reviewers. We strongly encourage code deposition in a community repository (e.g. GitHub). See the Nature Portfolio guidelines for submitting code & software for further information.

## Data

Policy information about availability of data

All manuscripts must include a data availability statement. This statement should provide the following information, where applicable:

- Accession codes, unique identifiers, or web links for publicly available datasets
- A description of any restrictions on data availability
- For clinical datasets or third party data, please ensure that the statement adheres to our policy

The data that support the findings of this study are not openly available due to reasons of confidentiality. Upon reasonable request, individual deidentified participant data (including data dictionaries) will be made available via a RedCap web-based database, after review and approval of a methodologically sound proposal, with a signed data access agreement, in line with Ethics Committee requirements. Please contact corresponding author, J.V. (joe.verghese@einsteinmed.edu). These files will be available from the date of publication until the date stated in the approved request. The study protocol is

available as an open access publication.

Data availability statement provided, page 28

## Human research participants

Policy information about studies involving human research participants and Sex and Gender in Research.

| | |
|---|---|
| Reporting on sex and gender | Primary outcome results were statistically significant in subgroups defined by sex, ethnicity, education, and language (Table 3). Sex was self-reported by participants. 865 (72.0%) were women, the rest were male.<br><br>Description provided in manuscript and in tables 1 and 3 |
| Population characteristics | Of the 1,201 participants with cognitive concerns, median age was 72.8 years (range 65-98), 865 (72.0%) were women, and 485 (40.4%) did not graduate high school. All participants (100%) resided in zip codes designated as socioeconomically disadvantaged neighborhoods (high Area Deprivation Index). 585 (48.7%) participants self-reported race as Black, and 667 (55.5%) ethnicity as Hispanic or Latino. 681 (56.7%) participants were assessed in English, and 520 (43.3%) in Spanish. Baseline characteristics were well balanced across arms (Table 1).<br><br>Description provided in manuscript and in tables 1 and 3 |
| Recruitment | Participants were recruited from one urban primary care clinic in Bronx County, New York, serving adults experiencing health disparities (underserved racial/ethnic minorities and residing in socioeconomically disadvantaged neighborhoods).1,3 All 18 primary care providers (16 physicians and 2 nurse practitioners) at this site participated in the trial. Research staff reviewed daily clinic schedules to identify potential participants, who were asked if either they or their loved ones were concerned about their memory function.1 Patients who answered affirmatively to either of the two cognitive concern questions were considered eligible for this trial (98.9%). A minority were recruited via patient self-referrals (0.4%) or staff referrals (0.7%).<br><br>Description provided in manuscript on page 23 |
| Ethics oversight | The Einstein institutional review board approved the study protocol.<br><br>Description provided in manuscript on page 23 |

Note that full information on the approval of the study protocol must also be provided in the manuscript.

# Field-specific reporting

Please select the one below that is the best fit for your research. If you are not sure, read the appropriate sections before making your selection.

☐ Life sciences ☒ Behavioural & social sciences ☐ Ecological, evolutionary & environmental sciences

For a reference copy of the document with all sections, see nature.com/documents/nr-reporting-summary-flat.pdf

# Behavioural & social sciences study design

All studies must disclose on these points even when the disclosure is negative.

| | |
|---|---|
| Study description | We conducted a single-blind RCT of the 5-Cog paradigm in primary care patients with cognitive concerns (NCT0381664). Data was quantitative in nature. Description in manuscript. |
| Research sample | Main inclusion criteria were age 65 years or more, presence of cognitive concerns, have a clinic appointment, and speak English or Spanish. Main exclusion criteria were prior dementia or mild cognitive impairment syndrome (MCI) diagnoses; nursing home resident, and inability to see or hear well enough to complete assessments. Full study criteria appear in our protocol paper. |
| Sampling strategy | Clinical trial at single primary care clinic site. |
| Data collection | Trained research assistants collected data from trial participants.<br><br>A trained outcomes assessor collected outcomes data blinded to randomized assignment. |
| Timing | 1,201 participants were enrolled from 29 May 2019 to 15 September 2022. Follow-up for the primary outcome ended on 15 December 2022, and on 15 May 2023 for the secondary outcome. Description provided in manuscript, page 4 |
| Data exclusions | No data from randomized participants was excluded. See figure 1 for overall sample. |
| Non-participation | In all, 9 participants withdrew from the study (5-Cog, n=7; control, n=2). Description provided in manuscript, page 4 |

| | |
|---|---|
| Randomization | 599 assigned to intervention and 602 to control. Description provided in manuscript, page 4 |

# Reporting for specific materials, systems and methods

We require information from authors about some types of materials, experimental systems and methods used in many studies. Here, indicate whether each material, system or method listed is relevant to your study. If you are not sure if a list item applies to your research, read the appropriate section before selecting a response.

## Materials & experimental systems

| n/a | Involved in the study |
|---|---|
| ☒ | ☐ Antibodies |
| ☒ | ☐ Eukaryotic cell lines |
| ☒ | ☐ Palaeontology and archaeology |
| ☒ | ☐ Animals and other organisms |
| ☐ | ☒ Clinical data |
| ☒ | ☐ Dual use research of concern |

## Methods

| n/a | Involved in the study |
|---|---|
| ☒ | ☐ ChIP-seq |
| ☒ | ☐ Flow cytometry |
| ☒ | ☐ MRI-based neuroimaging |

# Clinical data

Policy information about clinical studies

All manuscripts should comply with the ICMJE guidelines for publication of clinical research and a completed CONSORT checklist must be included with all submissions.

| | |
|---|---|
| Clinical trial registration | ClinicalTrials.gov number, NCT03816644 |
| Study protocol | Protocol paper cited. Paper included as supplementary material |
| Data collection | 1,201 participants were enrolled at a primary care clinic from 29 May 2019 to 15 September 2022. Follow-up for the primary outcome ended on 15 December 2022, and on 15 May 2023 for the secondary outcome.<br><br>Description also included in manuscript, page 4 |
| Outcomes | The primary outcome was improved dementia care actions related to diagnosis and management of cognitive impairment, a composite outcome met by documentation in EMR of any of the following within 90 days of visit: new PCP diagnoses of MCI or dementia; laboratory or imaging tests; new dementia medication prescriptions; or specialist referral for dementia evaluation. These dementia care actions are accepted as important by experts, and included in quality of care for dementia studies. The 90-day period was chosen as surveys in our site indicated that most dementia care actions were initiated within a week of the visit. The date of the action ordered and not the date completed was taken. Tests, referrals, or medications in our EMR can only be ordered by entering a medical indication. Investigations, referrals, or treatments were only counted as outcomes if a cognitive diagnosis (e.g., MCI) was entered as the indication. Any action ordered for non-cognitive medical indications were not counted towards outcomes. PCPs also made other cognitive diagnoses such as 'cognitive impairment,' 'cognitive deficit,' or 'memory loss,' in both the 5-Cog and control arms (6.7% vs. 3.7%, p=0.02). But these other cognitive diagnoses that do not have standard definitions were not a priori included in the diagnosis criterion for the primary outcome to minimize heterogeneity and ambiguity. However, they were considered as cognitive indications for the other endpoints (investigations, treatment or referrals) used to define the primary outcome.<br><br>Concerns that patients diagnosed with dementia have higher healthcare utilization and costs has been raised. Hence, healthcare utilization (emergency room visits and hospitalizations -any reason) up to 12 months after enrollment was examined as secondary outcome.<br><br>Description also included in manuscript, page 26 |
